# Caffeine and Primary (Migraine) Headaches—Friend or Foe?

**DOI:** 10.3389/fneur.2019.01275

**Published:** 2019-12-03

**Authors:** Karl B. Alstadhaug, Anna P. Andreou

**Affiliations:** ^1^Nordland Hospital Trust, Bodø, Norway; ^2^Institute of Clinical Medicine, The Arctic University of Norway, Tromsø, Norway; ^3^Headache Research, Wolfson CARD, Institute of Psychiatry, Psychology and Neuroscience, King's College London, London, United Kingdom; ^4^The Headache Centre, Guy's and St Thomas', NHS Foundation Trust, London, United Kingdom

**Keywords:** headache, caffeine, adenosine, dopaminergic, histaminergic, circadian, yawning, withdrawal

## Abstract

**Background:** The actions of caffeine as an antagonist of adenosine receptors have been extensively studied, and there is no doubt that both daily and sporadic dietary consumption of caffeine has substantial biological effects on the nervous system. Caffeine influences headaches, the migraine syndrome in particular, but how is unclear.

**Materials and Methods:** This is a narrative review based on selected articles from an extensive literature search. The aim of this study is to elucidate and discuss how caffeine may affect the migraine syndrome and discuss the potential pathophysiological pathways involved.

**Results:** Whether caffeine has any significant analgesic and/or prophylactic effect in migraine remains elusive. Neither is it clear whether caffeine withdrawal is an important trigger for migraine. However, withdrawal after chronic exposure of caffeine may cause migraine-like headache and a syndrome similar to that experienced in the prodromal phase of migraine. Sensory hypersensitivity however, does not seem to be a part of the caffeine withdrawal syndrome. Whether it is among migraineurs is unknown. From a modern viewpoint, the traditional vascular explanation of the withdrawal headache is too simplistic and partly not conceivable. Peripheral mechanisms can hardly explain prodromal symptoms and non-headache withdrawal symptoms. Several lines of evidence point at the hypothalamus as a locus where pivotal actions take place.

**Conclusion:** In general, chronic consumption of caffeine seems to increase the burden of migraine, but a protective effect as an acute treatment or in severely affected patients cannot be excluded. Future clinical trials should explore the relationship between caffeine withdrawal and migraine, and investigate the effects of long-term elimination.

“*I am not acquainted with any agents which equal these substances* (coffee and tea, aa), *in the power of removing headache without leaving inconvenient results. And as their physiological action is so purely cerebral, restoring the intellectual faculties, and ministering to the sensations of well-being, as well as lessening any sad emotions, we have here an adequate presumption, were any required, that this headache is seated in the nerves, which are immediately related with the molecular action of the brain.”*

John Addington Symonds, the Goulstonian lecture for 1858 (1)

## Introduction

It is well-know that caffeine can stimulate wakefulness, increase concentration and decrease the sensation of fatigue (2), but how does it affect one of the most common human agonies (3), headaches? Caffeine is commonly used as analgesic adjuvant for the acute treatment of pain. However, despite the flattering description of the efficacy above, the general analgesic effect of caffeine seems at best modest (4). Besides, chronic consumption of it may have a flip side, withdrawal may cause caffeine withdrawal (5, 6), a syndrome including symptoms such as drowsiness, headache, mood-changes, difficulty focusing, nausea and muscle pain/stiffness (Box 1). Even small amounts of caffeine have been shown to suppress this (5). Headache can occur independently of the other symptoms (7), and *Caffeine-withdrawal headache* (Box 2), properly described in the 1940s (8, 9), is recognized as an own diagnostic entity by the International Classification of Headache Disorders (ICHD-3) (10). Results from both experimental and clinical studies indicate a high rate of caffeine withdrawal in the modern society, that may even be underestimated (5). The real world extent and clinical (physiological and psychological) importance are not well-known (11). The dual effects of caffeine in headaches, relieving on one side and triggering on the other side, make caffeine a very interesting substance in headache pathophysiology research. Still, the prevailing theory of the withdrawal headache is basically a rebound vasodilation due to caffeine's vasoconstrictive effect (12), at large a too simplistic theory that is not in conformity with modern views of headache pathophysiology (13). The caffeine withdrawal syndrome, which includes symptoms suggestive of the prodromal phase of migraine, is hardly of peripheral origin. Based on the current established knowledge on migraine pathophysiology, this narrative review aims to explore how caffeine, which has profound biological effect as an adenosine receptor antagonist (14), may influence pathways involved in headaches, with a particular focus in migraine.

Box 1 **Diagnostic criteria for *Caffeine-withdrawal* according to the DSM-5.**

- Prolonged daily use of caffeine.
- Abrupt cessation of caffeine use, or reduction in the amount of caffeine used, followed within 24 h by 3 or more of the following symptoms:
  - Headache.
  - Marked fatigue or drowsiness.
  - Dysphoric or depressed mood, or irritability.
  - Difficulty concentrating.
  - Symptoms of nausea, vomiting, or muscle pain/stiffness.
- Clinically significant distress or impairment in social, occupational, or other important areas of functioning.
- Not due to the direct physiological effects of a general medical condition and are not better accounted for by another mental disorder.

Box 2 **Diagnostic criteria for *Caffeine-withdrawal headache* according to the ICHD-3.**

- Headache fulfilling criterion C.
- Caffeine consumption of >200 mg/d for > 2 weeks, which has been interrupted or delayed.
- Evidence of causation demonstrated by both of the following:
  1. Headache has developed within 24 h after last caffeine intake
  2. Either or both of the following:
    - Headache is relieved within 1 h by intake of caffeine 100 mg.
    - Headache has resolved within 7 days after caffeine withdrawal.
- Not better accounted for by another ICHD-3 diagnosis.

## Materials and Methods

The article is based on unsystematic searches in PubMed with terms like “caffeine and headache,” “caffeine withdrawal,” “adenosine and headache,” with more, and on own knowledge of older and recent literature on migraine. A discretionary selection of publications was made.

## Results and Discussion

### Caffeine and Adenosine

Caffeine is a major constituent of coffee and tea, but also naturally occur in guarana, cola nuts, cocoa, and several other plants (2). Soft drinks, energy drinks, and dietary supplements are also important sources, in particular among the younger population (15). The typical level in an ordinary cup of coffee varies between 50 and 100 mg (2). After oral ingestion, caffeine is rapidly and completely absorbed (99%), with peak plasma concentration reached usually within an hour (16, 17). Caffeine passes through all biological membranes, including the blood-brain barrier, and is distributed in all body fluids (18). It is metabolized by the cytochrome P450 system, the isoenzyme CYP1A2 responsible for 90% of caffeine clearance, and in adults it has a typical half-life of 5 h (19). Numerous factors, including a broad and variable genetic basis (20, 21), modify caffeine clearance.

The actions of caffeine in doses relevant to human consumption (50 to several 100 milligrams) are through antagonism of G protein-coupled purinergic (P1) receptors, more specifically the adenosine receptors (ARs), preferentially the A_1_R and A_2A_R (18, 22, 23). The human genes encoding for these two receptors are *ADORA*_1_ and *ADORA*_2_, respectively. Single nucleotide polymorphisms (SNPs) in *ADORA*_2_ appear to play a role in an individual's subjective response to caffeine (24–26).

Adenosine is found in every cell in the form of adenosine 5′-diphosphate (ADP) or adenosine 5′-triphosphate (ATP). It is continuously formed by breakdown of ATP and the physiological effects of adenosine are directly related to the metabolic activity (27). It modulates the activity of numerous cells, including mast cells, smooth muscle cells, platelets, and neurons (28). In the nervous system, adenosine appears to play an important role in modulating brain neurotransmitter release, locomotion, reward, sleep/wakefulness, cognition, and analgesia (14). Adenosine is neither stored nor released as a classical neurotransmitter, but may exert marked effects on neuronal excitability through G protein-coupled adenosine receptors (AR) A_1_, A_2A_, A_2B_, and A_3_ in both the peripheral and the central nervous system. The A_1_R is virtually found everywhere in the brain. In general it mediates tonic inhibition, especially through inhibition of glutamate release from presynaptic nerve endings (27). The A_2A_R is concentrated in dopamine-rich regions (18), especially on striato-pallidal GABAergic neurons (23), where it exerts excitatory effects on neurons and contributes to inhibition of motor activity (29). The A_2A_R actions are complicated by the fact that it co-localize and form heteromeres with dopamine-, cannabinoid-, and glutamate receptors (23). The roles of A_2B_R and A_3_R are not as well understood, but it appears that adenosine has a lower affinity for these receptors compared to A_1_R and A_2A_R receptors. Their activation is more likely to happen under conditions of hypoxia during which there is increased adenosine availability (30, 31).

### The Caffeine's Effects Are in General Opposite to the Effects of Adenosine

The brain levels of adenosine in cats and rodents in resting physiological condition have been estimated to 30–200 nM/L, concentrations sufficient to activate A_1_, A_2A_ and possibly A_3_ receptors if numerous on the cells (30, 31), but in most tissues the adenosine signaling is not very prominent (14). Even low concentrations of caffeine, such as 1–10 μM achieved after consumption of a single cup of coffee, result in significant antagonism of adenosine A_1_ and A_2A_ receptors and may result in increased alertness (32).

#### Sleep and Arousal

An important function of adenosine in the CNS is its involvement in the sleep/arousal system. Adenosine has sleep-promoting effects (33). During sustained and prolonged wakefulness the extracellular adenosine accumulates in the basal forebrain cholinergic region, and it declines slowly during recovery sleep (34). This rise of adenosine reduces the cortical activity by direct A_1_R modulation of the corticopetal system, the major extrathalamic relay of the reticular ascending system (RAS) to the cortex, and indirectly via A_2A_R-modulation of the hypothalamus (35). It has been shown, for instance, that infusion of a specific A_2A_R agonist into the subarachnoidal space, inducing NREM sleep, will cause increased activity in the ventrolateral-preoptic (VLPO) area of the anterior hypothalamus and a reduced activity of the tuberomammilary nuclei (TMN) in the posterior hypothalamus as seen by increased number of Fos-positive neurons (36). Caffeine-induced wakefulness depends on adenosine A_2A_ receptors (37). It seems that blocking of A_2A_R in nucleus accumbens inhibits the GABAergic output to the lateral hypothalamus, the TMN and the locus coeruleus (LC), causing activation and the major arousal effect of caffeine (38). Genetic knockout models have shown that both A_1_ and A_2A_ receptors are involved in mediating the sleep-promoting properties of adenosine in the brain (39). Moreover, the arousal effects of caffeine seen in wild-type animals are blunted in *ADORA*_2*A*_-knockout mice (38). It is therefore conceivable that adenosine receptor ligands could be used as normal cognitive enhancers or sleep promoters.

#### Pain

Experimental data indicate that caffeine at doses between 25 and 100 mg/kg have intrinsic antinociceptive effects (40). To extrapolate data derived from animal experiments to humans, it is generally assumed that giving 10 mg/kg caffeine to a rat corresponds to giving 3.5 mg/kg (about two to three cups of coffee) to a 70 kg human (18), although such an assumption may be premature given the higher metabolic rate of rodents and hence the shorter half-life of caffeine in their system. Based on this assumption, caffeine at doses around 600–1,200 mg may be needed to achieve anti-nociception in caffeine-naive humans. As an adjuvant caffeine (≥65 mg) can potentiate the analgesic properties of other medications (4, 41) by 40% (42), and there is a possibility that caffeine alone in such low doses might have intrinsic analgesic properties for some types of pain, such as headache. Ward et al. employed a double-blind placebo-controlled crossover design to assess whether caffeine alone (60 and 130 mg) has independent analgesic effects on non-migrainous headaches, and found equivalent effects to acetaminophen (43). Caffeine, and combination analgesics with caffeine may be used in tension-type headache, but frequent use is not recommended due to the risk of developing medication-overuse headache (41). It is also used in hypnic headache (44) and post-lumbar puncture headache (45), but probably not to the analgesic effect *per se*. In hypnic headache the effect of caffeine is claimed to go “beyond the usual analgesic effects observed in other headache disorders” (44). Hypothalamic effects, as described in the next section, may play a role. The effect on post-lumbar puncture headache, which has “not conclusive evidence” according to a Cochrane report from 2013 (45), has been attributed non-analgesic mechanisms, including adenosine-mediated vasoconstriction (46, 47).

Analgesic properties of caffeine may be hard to reconcile with analgesic effects of adenosine. When the concentrations of adenosine increase during stressful conditions, including noxious stimulation, it may reduce pain (48), and adenosine receptor agonists produce antinociception in a variety of pain models (49). Mice lacking A_1_R show signs of increased anxiety and hyperalgesia, and the antinociceptive effects of adenosine seen in wild-type mice (50) cannot be shown (48). A_1_R is present on peripheral sensory nerve terminals and in lamina II of the spinal cord, and it has been suggested that peripheral anti-nociception achieved by activation of A_1_R occurs via blocked release of endogenous calciton gene-related peptide (CGRP) (51) and substance P (52). Under inflammatory conditions, experimental data indicate that the analgesic effect is by reducing hypersensitivity through a central mechanism (49, 53). This is in accordance with human studies, showing that intrathecal injection of adenosine does not cause antinociception to acute thermal or chemical stimuli, but reduce allodynia from intradermal capsaicin injection (53). Adenosine may also increase pain mediated by A_2A_ receptors (28). Experimental data indicate that there are pro-nociceptive A_2A_Rs on the peripheral nerve terminals (28, 54), and that blocking of these may cause antinociception (40). Caffeine in doses normally consumed by humans can probably act as an analgesic, partly through blockade of A_2A_ receptor (40). Central dopaminergic mechanisms may also be involved. Like other analgesics (55), caffeine increases dopamine release (18), probably via inhibition of A_2A_R (56). Notably, A_2A_ receptor antagonists did not affect dural meningeal vasodilatation caused by CGRP in a rat model (57). The recent marketing of CGRP receptor antibodies and the development of CGRP antagonists has been considered a major breakthrough in the migraine field (58). Despite the fact that A_2A_R does not appear to be located in the spinal cord, a specific inhibition of the activity of the intermediolateral cell column (the sympathetic system) was shown by Brooks et al., indicating that the A_2A_R may be located on presynaptic inhibitory terminals of descending fibers from higher brain centers (59).

It is clear that both adenosine and blockade of adenosine by caffeine may cause anti-nociception. Since the nanomolar affinities of adenosine for A_1_R and A_2A_R are almost the same, this indicates a fine balanced modulation of the pain processing (30), making it very difficult to predict the net effects of caffeine on nociception in humans.

#### Caffeine Overuse and Withdrawal

Caffeine causes increased well-being in small to moderate doses and its overuse has the potential to cause physical dependence (60). The caffeine dependence syndrome has been recognized by the World Health Organization as a behavioral disorders due to frequent use of caffeine (61). Considerable evidence suggests that this is due to enhanced dopaminergic activity, especially via blocking of A_2A_R causing increased dopamine release in the ventral striatum (nucleus accumbens) (26). However, there is a compensatory up-regulation of the adenosine system, causing increased functional sensitivity to adenosine during withdrawal (5, 10). These molecular changes appear to increase functional sensitivity to adenosine during caffeine abstinence, and play an important role in the behavioral and physiological effects produced by caffeine withdrawal (5, 10). In humans, caffeine withdrawal following chronic consumption may give rise to a time-limited syndrome comprising of headache, drowsiness, mood-changes, difficulty focusing, nausea, and muscle pain/stiffness (Box 1) (5, 6, 62). Consequently, caffeine consumption may be maintained to avoid withdrawal symptoms. Caffeine-withdrawal headache is a headache developing within 24 h after regular consumption of caffeine in excess of 200 mg/day for more than 2 weeks, which has been interrupted. According to the ICHD3 classification, this type of headache resolves spontaneously within 7 days in the absence of further consumption (10).

Further, repeated exposure to caffeine may lead to rapid development of tolerance, preferentially to the A_1_-blocking effect, and in some cases it even may result in opposite effects than expected (14). By drinking three to four cups of coffee regularly around 50% A_1_ and A_2A_ receptor occupancy can be achieved for several hours, and many of the actions of caffeine are due to this AR blockade (18).

### The Migraine Syndrome

Migraine is a disorder characterized by recurrent attacks of head pain associated with hypersensitivity to sensory stimuli (10) and when full-blown it involves different phases (63). (a) A *prodromal or premonitory phase* hours prior to the onset of the headache with a broad range of symptoms (Table 1), which patients can reliably recognize, and thus predict the occurrence of a headache, (b) Transient neurological symptoms, known as *migraine aura* (typically visual alterations), just before the actual headache starts (in migraine with aura patients), (c) An intense *headache*, typically involving only one site of the head, accompanied by nausea, sensitivity to light, noise and smells, (d) The *postdrome phase* following the resolution of the headache and characterized mainly by fatigue and inability to concentrate (66). Understanding the mechanisms involved in the transition from a headache-free to the headache state is crucial in understanding the underlying cause of headaches and the development abortive drugs. In many primary headache disorders, but especially migraine (67, 68), several external factors have been reported to trigger this transition; stress, bright light and lack of sleep are probably the most commonly reported (69). The periodicity of migraine attacks strongly indicates involvement of internal clock mechanisms in its pathophysiology (70).

**Table 1:** Caffeine withdrawal symptoms and migraine prodromal symptoms.

**Frequent symptoms reported in different studies (underlined are the major criteria in the withdrawal syndrome)**	**Juliano (7)**	**Quintela (64)**	**Schoonman (65)**
	Caffeine withdrawal symptom, % (^*^)	**Migraine prodromal symptom, % (*n* = 100)**	**Migraine prodromal symptom, % (*n* = 461)**
Tiredness/fatigue/asthenia	21–56	31–38	47
Drowsiness/sleepiness	18–59	35 (“somnolence”)	NA
Difficulty concentrating	27–50	36	28
*Mood change*			
Depression/sadness	16^*^	39	18
Irritability	21	42	28
Yawning	21–43^**^	40	36
Nausea	3–33^***^	24	29
*Sensory hypersensitivity*			
Phonophobia	NR	37	36
Photophobia	14 (blurred vision^****^)	44	^*****^
Anxiety	10–29	46	NA
Craving	28–43^******^	15	17
Thirst	NR	17	NR
Muscle pain/stiffness	43	NA	35 (stiff neck)

* *Data were collected from 57 experimental and nine survey studies*.

** *21% reported in one survey and 43% in one experimental study. According to Juliano et al. (7) further research is needed to determine the validity of yawning as a withdrawal symptom*.

*** *3–21% in surveys and 10–33% in experimental studies*.

**** *Blurred vision was demonstrated in only 2 of 11 experimental studies*.

***** *Photophobia was excluded in this study*.

****** *Reported in 2 of 2 experiments. According to Juliano et al. (7) further research is needed to determine the validity of craving as a withdrawal symptom. NA, not assessed, NR, Not reported*.

#### Does the Migraine Prodromal Syndrome and Caffeine-Withdrawal Syndrome Share the Same Symptoms?

The prodromal, or premonitory, phase of migraine is usually defined as the period 2–48 h prior to aura or migraine headache with symptoms (Table 1) indicating an attack. Thirty to 90% of migraine patients report such a phase (71). In a prospective electronic diary study, Giffin et al. found that yawning, increased emotionality and concentration difficulties (difficulty reading and speaking) were the most reliable predictors (72). In another study by Schoonman et al., where patients had to choose among 12 specific premonitory symptoms, the most frequently reported were fatigue, phonophobia and yawning. Increased emotionality and concentration difficulties were reported by almost one third (10). Kelman found that tiredness, mood change and gastrointestinal symptoms (nausea) were the most frequent reported symptoms, and that yawning was rarely reported (69). However, there were specific questions about the former categories, but none about the latter symptom. In a prospective survey of 100 unselected patients, Quintela et al. (64) found that anxiety, phonophobia, irritability, unhappiness and yawning were the most common reported prodromal symptoms, whereas asthenia, tiredness, somnolence and concentration difficulties were the most common symptoms reported in the postdromal phase. Due to different methodologies used to identify symptoms, it may be difficult to compare results from different studies, but interestingly, the most prevalent symptoms reported are much the same as the symptoms of the caffeine-withdrawal syndrome. In Table 1 findings from the very good and comprehensive review about caffeine withdrawal symptoms made by Juliano et al. (7) are compared with findings from two of the mentioned surveys. These data strongly suggest that the same or similar pathophysiological pathways may be involved in both the prodromal phase of migraine and the caffeine withdrawal syndrome. However, sensory hypersensitivity, a cardinal migraineous feature (10), does not seem to be part of the caffeine withdrawal syndrome. This may nevertheless be due to an underlying thalamocortical dysrhythmia (73), that appears to be specific for migraine. The overlap of symptoms may also be due to the fact that caffeine withdrawal may act as a trigger of a migraine attack in migraine patients?

#### Is the Caffeine Withdrawal Headache Similar to Migraine?

Headache as a caffeine withdrawal symptom has been frequently studied (7), and in some case reports and experimental studies it has been characterized. However, premorbid primary headache syndromes, even in otherwise solid papers (74), are seldom reported. It seems though that subjects with a history of frequent headaches (75) are at increased risk, and that migraineurs and subjects who experience withdrawal headache share some comorbidity such as major depression and anxiety (5). To “produce experimental headaches which would be more physiological than experimental histamine and nitrite headaches,” Dreisbach in 1940 gave a number of non-habitual coffee drinkers capsules of 10–12 grains (650–780 mg) of caffeine daily for 1 week and then withdrew the treatment. On the day of withdrawal, almost all developed a moderate to severe headache. In migraineurs, “typical migraine syndromes” ensued (8). Unfortunately, the report does not give sufficient data for valid interpretation. Three years later, however, he and a colleague reported 38 similar trials in 24 persons of whom 5 had migraine. In 21 of the trials the subjects experienced their worst headache ever, in 11 it was definite but not severe, and in 6 there was slight or no headache. All of the migraineours experienced caffeine withdrawal headache “quite different from the migraine syndrome.” It is reason to question this statement. The headache was accompanied by nausea in 4 and vomiting in 1. In 4 subjects the headache was consistently accompanied by serous rhinorrhea. This may indicate cranial autonomic symptoms, frequently seen in migraine (76). No information about other accompanying symptoms was given. It was argued that 2 subjects had headache localized to the opposite side of the head from their usual migraine. In one subject, who used to have left-sided frontal migraine, it occurred bilaterally and was localized in the occipital region. Our clinical experience, however, confirms that is not unlikely for migraine to switch sides or to be bilateral rather than unilateral. Scotomas, regularly experienced by 3 migraine patients, did not occur. Scotoma, is a symptom usually described in migraine with aura patients and to date no headache-induced substance has been found to consistently trigger migraine aura. Further, two of the migraineurs had migraine attacks on the height of the caffeine stimulation, and in one of these subjects this reoccurred in a second trial. In these trials, and in later studies the headache is described to typically evolve gradually (60, 77, 78), being diffuse (79), throbbing (9), severe (60, 80), intensified with exercise (77), and Valsalva manoeuver (9) and having a mean duration for 2–3 days (81).

Despite being claimed that caffeine-related headache has a non-migrainous clinical presentation (82), the description of caffeine withdrawal headache given in the literature is not very different from migraine. With the high prevalence of migraine (83), and the lack of information on pre-existing headache in many studies taken into consideration, there seems to be a clear shortcoming in the knowledge when it comes to separating caffeine withdrawal as a migraine trigger from caffeine withdrawal headache *per se*.

#### Does Caffeine Cause Headaches?

Caffeine itself is rarely reported as a trigger of migraine (68), but as noticed by Driesbach it probably has the potential (9). As an important cause of *medication-overuse headache* (84) caffeine has been strongly incriminated, partly due to the withdrawal syndrome encouraging patients to continue their overuse (85–87). In a double blinded, randomized, placebo-controlled, 12 week crossover study of 45 healthy subjects who habitually consumed 4 to 6 cups of coffee a day (81),withdrawal caused headaches during the first or second day in 19 (42%) (81). The ratio of subjects reporting headaches during caffeine weeks and during non-caffeine weeks was 1. However, what is obvious, but not commented by the authors, is that when the withdrawal weeks (week 1 and 7) are removed the ratio increases to 7.7. Subjects also reported improved sleep during the non-caffeine weeks.

Caffeine-overuse may even cause chronic headache in children (15). Hering-Hanit and Gadoth reported 36 children with chronic headache, none with a prior history of migraine, who consumed excessive amounts of caffeine in the form of cola drinks (in average ~190 mg caffeine/d), and of whom 33 completely recovered after gradual withdrawal. Three children continued to have infrequent migraine without aura after withdrawal (15). In general, most chronic daily headaches in children appear to be migraine related (88). Caffeine has also been a suspected risk factor for episodic migraine evolving into *chronic migraine*, which in essence is defined as headache on ≥15 d/month for >3 months (66). A strong positive correlation between caffeine consumption and both episodic (89) and chronic migraine (90) has been found. In a retrospective population-based study, Scher et al. found that dietary and medicinal caffeine consumption appears to be only a modest risk factor for developing chronic daily headache, including chronic migraine (91). Overall, no relationship between current caffeine consumption and headache was found, neither was that found in two further studies (92, 93). However, in the cross-sectional population-based study by Boardman et al., headache sufferers were more than twice as likely to be heavy caffeine consumers compared to non-sufferers (92). In a Norwegian large-scale cross-sectional population-based study only a small association between high consumption of caffeine (>540 mg/d) and infrequent migraine was found (94). In this study chronic headache was more prevalent among individuals with low caffeine consumption (mean of 125 mg/d).

In general, chronic consumption of caffeine seems to be associated with increased migraine burden. Either chronic caffeine increases the risk of migraine *per se*, or in contrast, has beneficial effects in patients severely affected by migraine. Whether long-term elimination of caffeine diet will reduce the migraine burden has not been studied previously.

### Pathophysiological Aspects

In 1990, Welch et al. substantiated the concept of migraine as a state of central neuronal hyperexcitability (95). Later it has become clear that some sort of cortical dysexcitability may even be present between attack (73). The mechanisms underlying the abnormal regulation of cortical function and the cyclic features of migraine remain largely unknown, but older, largely intuition based theories of hypothalamic dysfunction as the cause of periodicity of attacks have in recent years had a renaissance, especially after brain imaging evidence of hypothalamic activity both in the prodromal (96) and the pain phase (97) of migraine. There is accumulating indirect evidence for a pivotal role of hypothalamus in many primary headache disorders (98–102). The discovery of a mutation in a clock-gene (CK1δ) causing so called familiar advanced sleep phase syndrome was strongly linked to migraine both clinically and experimentally (103). Mice engineered to carry the mutation exhibit lower threshold for cortical spreading depression (CSD), the phenomenon underlying aura (see beneath), and increased sensitivity to noxious stimuli after nitro-glycerine treatment. A clear relationship between sleep and migraine exists (104), and caffeine can certainly disrupt sleep (105). Disrupted sleep-patterns predispose, and sleep *per se* protects against migraine (100). The risk of getting a migraine attack is low during sleep, but spikes in the morning, especially when associated with insomnia (106), and peaks in the afternoon probably due to work-related stress (107). Menstrual migraine and weekend headaches are also clear examples of the periodicity. The higher prevalence of migraine on weekend mornings reported was attributed to caffeine withdrawal by Couturier et al. (108).

#### Adenosine Can Cause Headache

It is now accepted that the pain during a migraine attack is perceived to be felt on intracranial structures, such as, the dura mater and intracranial vasculature (109, 110). Activation of the ophthalmic division (V1) of the trigeminal nerve is regarded primarily responsible for causing the pain in primary headache disorders (102). Upon activation of the trigeminal fibers, several neuropeptides such as substance P, neurokinin A, calcitonin gene-related peptide (CGRP), pituitary adenylate cyclase activating peptide (PACAP) and nitric oxide (NO) are released into the innervated tissue (dura mater and meningeal vessels) with the potential to cause neurogenic inflammation. Migraine has been attributed to such a local sterile meningeal inflammation (111), possibly involving release of histamine from mast cells (112). Activation of the trigeminal fibers is referred to as “trigeminovascular activation” and is considered the key event in causing headache. Accumulating data support a pivotal role of CGRP in migraine (58), and when given intravenously a delayed migraine-like headache will be induced in a large fraction of migraineurs but not in controls (113). CGRP causes only a modest, around 10%, vasodilation which is unlikely to activate perivascular trigeminal afferents (114). In general, the former “vascular theory” of migraine has been abandoned by several experiments showing that vasodilatation of neither extracranial- or intracranial/meningeal arteries is neither necessary nor sufficient to cause migraine headache (113). However, vasodilatation may worsen pain in an already sensitized pain network. The trigeminal fibers, that carry the sensory information from the intracranial structures, project on second-order neurons within the trigeminocervical complex (TCC; trigeminal nucleus caudalis, C1 and C2 spinal levels). These neurons give rise to the main ascending trigeminothalamic pathway that carries sensory information to third order neurons in the thalamus, before processing the information to higher cortical areas (115).

As with other recognized experimental triggers of migraine (116), such as CGRP, it is hard to conceive how adenosine itself, not passing the blood-brain barrier readily (14), could act centrally to elicit a migraine cascade. Further, adenosine has been ascribed peripheral anti-nociceptive effects by blocking the release of CGRP (51). Nevertheless, increased levels of adenosine in the blood during migraine attacks have been reported (117), and headaches are often reported when adenosine is used intravenously in cardiology (118). The A_2A_ agonist regadenoson does also induce headache frequently (119). The drug dipyramidole, which increases the level of adenosine by inhibiting adenosine re-uptake, can cause headache as an adverse effect in one third of the patients. It has been also reported to increase the migraine frequency in migraine patients (120) and to elicit migraine in an experimental setting (121). In addition, intravenous application of adenosine can trigger migraine attacks (122). Based on this, antagonizing adenosine by caffeine may theoretically have an abortive effect in migraine. The problem is, as mentioned earlier, that chronic caffeine consumption in humans seems to increase the migraine burden. Supported by experimental findings (123, 124), an increased vascular tone due to up-regulation of adenosine receptors and compensatory increased levels of plasma adenosine have been suggested mechanisms underlying this risk (125, 126). Increased cerebral blood velocities have been measured after caffeine withdrawal (12), and there is no doubt that caffeine has the opposite effect causing a decrease in cerebral blood flow due to central vasoconstriction. However, the neuronal-vascular coupling is complex and in a functional magnetic resonance imaging study (fMRI), reduced cerebral perfusion induced by caffeine was independent of previous caffeine consumption (127). In a study of the rat middle meningeal artery, caffeine was found to reverse the relaxing effect of adenosine, mainly mediated by blocking of A_2A_R (128). Whether vasoconstriction significantly contributes to the effects of caffeine in withdrawal headache remains unclear since vasodilation hardly is a primary cause of the headache. As mentioned earlier, blocking of A_2A_R does not seem to affect neurogenic vasodilation (57). Further, discrepancy between different studies has been observed. In an experimental cat migraine model, the A_1_R agonist GR79236 had a dose-dependent inhibitory effect on trigeminovascular nociceptive transmission (129). This effect was explored in humans. In 12 healthy female volunteers the adenosine A_1_ receptor agonist GR79236 was shown to inhibit trigeminal nociception, as measured by the blink reflex (130). Further studies in pigs did not show any effect on capsaicin-induced CGRP release (131), and in a multicenter evaluation of the adenosine agonist GR79236X in patients with dental pain after third molar extraction, no efficacy was shown when compared to placebo (132).

#### Caffeine Has the Ability to Induce Cortical Excitability, and May Even Predispose for CSD?

Traditionally, the migraine aura has been considered a distinctive phase, but probably it is a consequence of the same or parallel mechanisms that triggers the pain (133, 134). It is believed that the migraine aura itself is caused by so called cortical spreading depression (CSD) (135). CSD is a wave of neuronal depolarisation linked with depressed neuronal activity and blood flow changes (136). In animals, CSD can quite readily be induced by focal cortical stimulation for example by applying K^+^ (137). Despite being accepted by most experts as a plausible mechanism of migraine aura and even headache in migraine without aura (“silent spreading depression”) it has been difficult to prove that CSD actually is the underlying mechanism in humans. It also remains enigmatic how CSD could be triggered in patients during migraine. So called calcium waves in astrocyte networks is a speculative (138), but alluring mechanism that could offer an alternative explanation to the classical CSD. Recent advances support the idea that astrocytes could play an important role in spreading depression initiation (139). ATP-receptors (P2 receptors) are both necessary and sufficient for propagation of calcium waves (140), and thus has the potential to initiate and sustain a heighten state of neuronal excitability. Caffeine may possibly modulate this susceptibility (141). An A_2A_ receptor gene haplotype has been reported to be associated with migraine with aura (142), but the findings should be reproduced. There appears to be only one published case where intravenous adenosine precipitated migraine aura (122). Activation of A_1_R has been shown to increase K^+^ conductance and thus hyperpolarize CNS neurons (50). It is thus more plausible that acute caffeine could increase the susceptibility to CSD, and pre-published reports supported that (143, 144). However, caffeine exposure did not affect the susceptibility to CSD in a recent study of mice (82). Curiously, CSD can induce yawning in rats (145).

#### Yawning Indicates Hypothalamic Alterations

It has been demonstrated that activating the A_1_R on TMN-neurons increases NREM sleep (146), and that blocking them on hypocretinergic neurons of the lateral hypothalamus increases wakefulness (147). Both neurotransmitter systems have been suggested to play an important role in migraine (148, 149). The TMN of the posterior hypothalamus has been suggested to play a role in the initial phases of a migraine attack and to be responsible for the morning occurring migraine attacks (148, 150). During drowsiness and normal recovery sleep the firing from the histaminergic neurons are reduced or absent, but during wakening and arousal they fire, allegedly the most wake-selective firing pattern identified to date (151). Adenosine may well have a protective effect against migraine during sleep, but during non-recovery sleep and wakefulness disrupted homeostasis may cause increased histaminergic firing predisposing for headaches. It has been postulated that yawning is the manifestation of a switch in brain states from “default mode” to an “attentional mode” by increasing clearance of adenosine (152). It remains to be proven in experimental models that increased histaminergic firing sensitizes the TCC.

Yawning may also be indicative of an individual's inability to properly maintain thermal brain homeostasis (153). If yawning occurs without being associated with tiredness, it may perhaps indicate a thermoregulatory dysfunction. In the study of Schoonman et al. of the premonitory symptoms of migraine there was no correlation between “sleep problems” and yawning (Spearman's rank correlation of 0.024) (65). Further, Jacome described 3 migraineurs with compulsive yawning as a prodromal symptom, independent of fatigue and drowsiness (154). In a recent cross-sectional study, 45.4% of 339 migraineurs reported repetitive yawning during migraine attacks (155). Sleepiness was significantly more often reported in patients with yawning compared to those who did not yawn during their migraine attacks.

Thermoregulation and sleep are interrelated. It is well-known that yawning has a clear circadian pattern parallel to the rise in body and brain temperature, normally occurring most often before sleep onset and after waking (153). A hypothesis that migraine attacks serve to restore the brain temperature has recently been put forward (156). In general, the neurons of the brain are very sensitive to variations in the temperature (157). Short visual stimulation of the rat invokes a rise in temperature over the visual cortex (158), and during prolonged (4 min) visual stimulation in man, an increase in regional cerebral blood flow caused an average decrease in temperature by 0.2°C (159). Histamine has been shown to mainly excite heat-sensitive neurons in the anterior hypothalamus, causing hypothermia. In contrast to adenosine (160), caffeine increase body temperature parallel to arousal during circadian misalignment in humans (161). Injecting neuropeptide orexin-A into the rat PVN elicits a cortical arousal response followed by yawning (162), and injecting it into the ventromedial hypothalamus it induces hyperthermic reactions (163).

Based on the fact that migraineurs show a lower threshold for central dopamine receptor activation than normal subjects (164), and that exogenously administered dopamine receptor agonists may produce some symptoms experienced in the prodromal phase of migraine such as drowsiness and yawning, dopamine may play an important role in migraine pathophysiology. This theory is consistent with the idea that caffeine withdrawal symptoms are due to increased sensitivity of adenosine, causing increased drowsiness (due to increased disinhibition of VLPO sleep-active neurons reducing histaminergic tone) and excessive yawning due to increased dopaminergic tone. It has been shown that injecting dopamine (D2) agonists into the paraventricular nucleus of the hypothalamus (PVN) of rats, increases local nitric oxide (NO) production and thereby activates central oxytocinergic neurotransmission, inducing yawning (165). However, microinjection of other substances into the PVN, such as histamine (166) and nitroglycerine (167) also induces yawning. As distinct from dopamine agonists, that seldom induces headache (168), both donors of NO and histamine are established triggers in pharmacological models of migraine (116). Glyceryl trinitrate has even been shown to both induce prodromal symptoms of migraine (169) and activate the hypothalamus (96).

#### Caffeine Can Alter the Circadian System and Neuronal Excitability

It has been proposed that the hypersensitivity to light during migraine may be exerted by intrinsically photosensitive retinal ganglion cells (ipRGCs) through a pathway that modulates the activity of dura-sensitive thalamocortical neurons (170). The ipRGCs entrain the circadian rhythms and influence sleep/wakefulness (171). Adenosine, through A_1_R inhibition of glutamate release, seems to fine-tune the circadian system through gating of both photic and non-photic input to the superior biological clock, the suprachiasmatic nucleus (27). Giving an A_1_R agonist during midday, phase-shift mimicking the effect of a 3 h sleep deprivation procedure may be achieved in hamsters (172). Caffeine increases light responsiveness of the mouse circadian pacemaker (173), and it has been shown that chronic exposure to caffeine interferes with the ability of the SCN to entrain normally to light, and that it potentiates phase-delays (174). Further, it has been claimed that glasses that filter out blue light can reduce the frequency of migraine attacks with short periods of usage (175), and in an experimental study light stimulation with the peak wavelength of ipRGC induced migraine attacks more frequently than extensive-wavelength (176). Based on this, it may be speculated that caffeine interferes with the ability of the biological clock to entrain to light, causing increased excitability in pathways involved in migraine headache, including the dura-sensitive thalamocortical neurons.

The pharmacology of both the TCC and the thalamus provides interesting insights into migraine pathophysiology, as they are prominent sites of action of migraine specific medication-triptans (177), of clinically active preventives (178, 179) and of other potential anti-migraine compounds (180). The modulation of sensory transmission in the thalamus, assumes further significance as it has been shown not only to be a pivotal area for the development of sensory hypersensitivity to light during migraine (170), but could also participate in the development of hypersensitivity to noise (181), and non-cranial allodynia that is frequently seen in migraine patients (182). Experimental data show that presynaptic adenosine A_1_ receptors on thalamo-cortical neurons can mediate reduced cortical excitability directly (183). When interacting with serotonin, adenosine may also modulate thalamic sensory gating during sleep (184). If the caffeine withdrawal syndrome is due to increased sensitivity to adenosine, this may explain why sensory hypersensitivity is not a symptom of caffeine withdrawal. It is tempting to speculate that caffeine can increase the sensory hypersensitivity that accompanies migraine headache.

Both the TCC and the thalamus have reciprocal direct or indirect connections with multiple brainstem, midbrain and cortical nuclei that control the excitability of the ascending trigeminothalamic pathway (185). These brain nuclei make up the descending pain modulatory system (115), which is a powerful regulator of pain-related activity along the ascending trigeminothalamic pathway. Disruption of normal endogenous descending modulatory tone may play a critical role in primary headache disorders, but what really alters the excitability of the ascending trigeminothalamic pathway in a manner that a migraine attack develops in susceptible individuals, remains to be revealed. As mentioned earlier, central dopaminergic mechanisms (via A_2A_R) may be involved. In an experimental headache model, stimulation of the dopaminergic A11 significantly inhibited peri-MMA dural and noxious pinch evoked firing of neurons in the TCC, an effect that was blocked by a D2 receptor antagonist (186). Whether the A11 neurons have adenosine receptors, and whether caffeine induces release of dopamine from the A11 nucleus, is not known.

## Conclusion

The current opinion is that caffeine both can relieve and trigger headaches. It has to be clarified whether caffeine withdrawal triggers or merely resembles the migraine syndrome. The nature of the caffeine withdrawal syndrome needs to be better understood. In assessing the clinical effects of caffeine withdrawal, there is a chance that a triggered migraine syndrome is interpreted as part of the caffeine withdrawal syndrome, explaining an overlap between these two. If it triggers migraine it offers a good human migraine model. A clinical trial thoroughly evaluating the withdrawal syndrome specifically in subjects with migraine should be performed. Furthermore, whether long-term elimination of caffeine diet will reduce or increase the burden of migraine should also be evaluated.

There is compelling evidence that adenosine may trigger headaches, but how this poor blood-brain-barrier penetrating substance can trigger central mechanisms when given intravenously remains enigmatic. Chronically blocking adenosine receptors by habitually drinking coffee seems to increase the burden of migraine, and it is tempting to believe that this causes an increased sensitivity to adenosine, evident when caffeine is withdrawn. Caffeine withdrawal and migraine prodromal symptoms are definitely caused by alterations in the CNS. Looking beyond the peripheral effects, central adenosine mechanisms should be explored in experimental headache models. The link between adenosine, the circadian system, sleep, and pain points at the posterior hypothalamus as a locus in quo. The effects of caffeine on the TMN and the A11 may offer novel insight, and the A_2A_R seems to be of particular interest. Two positron emission tomography (PET) A_2A_R-ligands have been developed, and this may render human *in vivo* imaging studies possible (14). Future research should also confirm and investigate a role of receptor genes like ADORA2A in migraine and caffeine withdrawal.

## Author Contributions

AA conceived the manuscript. KA wrote it with support from AA.

### Conflict of Interest

The authors declare that the research was conducted in the absence of any commercial or financial relationships that could be construed as a potential conflict of interest.
